# A Comparison of the Nutritional Quality of Food Products Advertised in Grocery Store Circulars of High- *versus* Low-Income New York City Zip Codes

**DOI:** 10.3390/ijerph110100537

**Published:** 2013-12-31

**Authors:** Danna Ethan, Corey H. Basch, Sonali Rajan, Lalitha Samuel, Rodney N. Hammond

**Affiliations:** 1 Department of Health Sciences, Lehman College, The City University of New York, 250 Bedford Park Boulevard West, Gillet Hall, Room 334, Bronx, NY 10468, USA; E-Mail: danna.ethan@lehman.cuny.edu; 2 Department of Public Health, William Paterson University, Wing 150, Wayne, NJ 07470, USA; E-Mail: Baschc@wpunj.edu; 3 Department of Health and Behavior Studies, Teachers College, Columbia University, 525 West 120th Street, New York, NY 10027, USA; E-Mail: sr2345@tc.columbia.edu; 4 Department of Health Sciences, Lehman College, The City University of New York, 250 Bedford Park Boulevard West, Gillet Hall, Room 421B, Bronx, NY 10468, USA; E-Mail: lalitha.samuel@lehman.cuny.edu; 5 Department of Health and Nutrition Sciences, Montclair State University, 1 Normal Avenue, Montclair, NJ 07043, USA; E-Mail: rodneyh8890@gmail.com

**Keywords:** New York City, grocery store circulars, promotional strategies

## Abstract

Grocery stores can be an important resource for health and nutrition with the variety and economic value of foods offered. Weekly circulars are a means of promoting foods at a sale price. To date, little is known about the extent that nutritious foods are advertised and prominently placed in circulars. This study’s aim was to compare the nutritional quality of products advertised on the front page of online circulars from grocery stores in high- *versus* low-income neighborhoods in New York City (NYC). Circulars from grocery stores in the five highest and five lowest median household income NYC zip codes were analyzed. Nutrition information for food products was collected over a two-month period with a total of 805 products coded. The study found no significant difference between the nutritional quality of products advertised on the front page of online circulars from grocery stores in high- *versus* low-income neighborhoods in New York City (NYC). In both groups, almost two-thirds of the products advertised were processed, one-quarter were high in carbohydrates, and few to no products were low-sodium, high-fiber, or reduced-, low- or zero fat. Through innovative partnerships with health professionals, grocery stores are increasingly implementing in-store and online health promotion strategies. Weekly circulars can be used as a means to regularly advertise and prominently place more healthful and seasonal foods at an affordable price, particularly for populations at higher risk for nutrition-related chronic disease.

## 1. Introduction

Research indicates that the built environment impacts quality of health. In the area of nutrition, it is clear that communities rely on what is locally and readily available when making food choices for themselves and their families [1,2]. Recent public health reports have highlighted the problem of inadequate availability of nutritious foods in low-income neighborhoods as well as for ethnic minorities [3,4]. Less access to healthy whole foods such as fruits and vegetables can increase risk of developing chronic disease, particularly those comorbidities associated with obesity [5,6]. Conversely, greater access to nutritious foods has been linked to lower rates of obesity. For instance, the presence of—and residents’ proximity to—neighborhood grocery stores has been linked to a decreased risk of obesity, lower rates of diabetes, and better eating habits [7,8,9,10]. 

Grocery stores can be an important resource for health and nutrition, namely given the wide variety and economic value of foods that are available [4,11]. These food establishments also have the opportunity to positively influence customers’ habits of purchasing more healthful foods. An increasing number of grocery stores across the country are launching wellness programs based on customer demand for more accurate information on and availability of nutritious foods [12,13]. Other health-promoting means utilized by certain grocery stores include coupons and promotional sales that are advertised in weekly circulars [14]. 

In a recent study, food products advertised on the front page of circulars from grocery stores located in urban, low-income neighborhoods were found to be mostly processed, high in carbohydrates, and low in fiber [15]. The circulars also offered a paucity of fresh fruits and green leafy vegetables; packaged foods were often high in sodium and saturated fat [15,16]. 

These findings, among others, highlight the problem of affordable access to nutritious foods in urban neighborhoods with high rates of diabetes and obesity [17,18,19]. In general, higher-income areas offer greater access to healthful foods, providing greater quantity, better quality, and more variety [4]. To date, little is known about how often these foods appear in promotional materials such as circulars. As such, this study’s aim was to compare the nutritional quality of food products advertised on the front page of circulars from grocery stores in high- *versus* low-income neighborhoods in New York City. We hypothesized that the grocery stores from low-income zip codes would advertise a greater number of food products that were lower in nutritional value.

## 2. Experimental Section

### 2.1. Sample Selection

Prior to sample selection, we first determined median household income for all zip codes in New York City using U.S. Census data [20]. The study’s sample consisted of grocery stores from the five zip codes representing highest median household income with grocery stores present and five zip codes representing the lowest income in this category. To identify grocery stores in these zip codes, we accessed three separate, online search engines (for cross-checking purposes) and entered the terms, “grocery stores” and “supermarkets” by zip code in these two income groups. If there were no grocery stores located in a zip code that was entered, the next highest (or lowest) median household income zip code was entered. Only grocery stores with online, weekly circulars were eligible for selection. Once stores with online circulars were identified for both zip code groups, one store per zip code was randomly selected for a total of ten sites. All stores selected for the high-income group were located in Manhattan. Among stores in the low-income group, three were located in the Bronx and two in Brooklyn. It should be noted that all ten grocery stores were part of larger grocery chains.

### 2.2. Data Collection

Nutritional information for all food products advertised on the first page of online circulars in both the high- and low-income groups was collected every other week from May to July, 2013. Each advertised food product was entered into the United States Department of Agriculture’s (USDA) National Nutrient Database for Standard Reference Coding Chart and categorized into one of the 25 existing food groups [21]. For instance, the database categorized pizza as a “fast food” and ice cream as a “sweet.” All products listed in the beverage category were removed from the data set for separate analysis. 

For each food product, nutrient information was recorded from the nutrition facts label on the manufacturer’s website. When such information was missing online, it was obtained directly from the physical label. In the case of unbranded foods and produce, nutrient information was obtained from the USDA Nutrient Database. The nutrient information included amount of carbohydrates, fiber, and sodium present in one serving. 

Foods with at least one carbohydrate choice per serving were identified as those that contained a minimum of 15 grams of carbohydrates per serving [22]. Reflecting the federal guidelines for nutrition facts labeling, high-fiber foods were identified as those with 5 grams or more of fiber per serving, and products containing less than 120 milligrams of sodium per serving were categorized as low in sodium [23,24]. Products advertised as reduced-, low-, or zero-fat in the circulars were also coded as such. This study was determined to be exempt/not human subjects research by the Institutional Review Boards at Lehman College, William Paterson University, and Teachers College, Columbia University. 

### 2.3. Data Analysis

Circulars were analyzed across the aforementioned 10 grocery stores in New York City. All data were organized and analyzed in SPSS (version 20.0, IBM Corp., Armonk, NY, USA). Within each food group, the proportion of foods with specific nutritional or product types was calculated. These included: (1) baked products, breakfast cereals, cereal grains and pasta products that contained at least one carbohydrate serving and/or were high in fiber, (2) products within the “fruit and fruit juices” food group that could be categorized as fresh fruits, fruit juice and canned or frozen fruits, and (3) products within the “vegetables and vegetable products” food group that could be categorized as fresh, canned, or frozen vegetables [15]. The proportion of products with the aforementioned characteristics within each USDA category that were promoted by multiple sales was also calculated. A “multiple sale” encouraged customers to purchase multiple units of the same product by advertising prices such as “buy one, get one free,” “buy two for $3.00,” or multiple units packaged together, such as a pack of six soda cans. 

Further, z-ratios were calculated to determine if the independent proportions of food products within specific USDA categories were significantly different between the low- and high-income groups [25]. In addition, independent sample t-tests were run to determine whether the number of products promoted in the circulars and nutritional quality of products differed significantly between the low- and high-income zip code groups. Chi-square statistics were also utilized to look at differences in types of promotions across low- *versus* high-income zip codes. A total of 805 products featured in the circulars were identified across the entire sample. A total of 288 products were advertised in circulars from the high-income group *versus* 517 products in the low-income group.

## 3. Results and Discussion

Online circulars from ten New York City grocery stores were analyzed. Five of the stores were located in the zip codes with the highest median income where grocery stores were present (all located in Manhattan); the remaining five were present in the lowest median income zip codes (three Bronx-based and two in Brooklyn). Of the 805 products identified in the circulars, 288 were advertised in circulars from the high-income group *versus* 517 products in the low-income group’s. Results confirmed that the mean number of products per store was statistically significantly different between low- and high-income zip codes (*t* = 2.533, *p* < 0.05). Specifically, the grocery stores in the low-income group promoted significantly more products as compared to those in the high-income group.

### 3.1. Comparison of Products and Nutritional Content

All food products on the circulars’ front page were classified using the USDA’s Nutrient Database for Standard Reference Coding Chart which allowed for a consistent and standardized coding process [21]. An in-depth analysis of the nutrition content of all advertised foods was also conducted. In several instances, products met the characteristics of more than one USDA category and were therefore coded as such. For example, a product may have been categorized as a snack and also noted as containing at least 15 grams of carbohydrates per serving. The data presented therefore reflect these details. Table 1 presents the frequency distribution and corresponding proportion of featured circular products comprising each USDA category. The proportion of products that met specific nutrition characteristics is also described. Specifically, the proportion of products featured in the USDA categories of “fats and oils, spices and herbs, soups, sauces and gravies” (z = −4.19, *p* < 0.001), “fruits and fruit juices” (z = 3.81, *p* < 0.001), and “breakfast cereals, cereal grains and pasta products” (z = −3.85, *p* < 0.001) were found to be significantly different. Circulars from high-income grocery stores promoted more fruits and fruit juices and those from the low-income group advertised more products representing the remaining two groups.

**Table 1:** Frequency distribution of featured circular products: low- *versus* high-income grocery stores in New York City

Products	High-Income (N = 288)	Low-Income (N = 517)
Processed foods	66.7% (n = 192)	69.8% (n = 361)
Foods containing at least 15 g of carbohydrates/serving	24.0% (n = 69)	27.1% (n = 140)
Foods containing at least 5 g of fiber/serving	4.5% (n = 13)	3.5% (n = 18)
Foods advertised as reduced-/low-/zero-fat	0.3% (n = 1)	0% (n = 0)
Foods containing low sodium levels	0% (n = 0)	0% (n = 0)
Breakfast cereals, cereal grains and pasta products	1.4% (n = 4)	8.5% (n = 44)
Beef, poultry, lamb, veal, pork, sausage, luncheon meats, fish and shell fish products	26.4% (n = 76)	20.1% (n = 108)
Dairy and egg products	10.1% (n = 29)	9.3% (n = 48)
Fast foods, meals, entrees, side dishes and restaurant foods	2.1% (n = 6)	1.7% (n = 9)
Fats and oils, spices and herbs, soups, sauces and gravies	3.3% (n = 9)	11.8% (n = 61)
Fruits and fruit juices	20.1% (n = 58)	10.4% (n = 54)
Legumes and legume products, nut and seed products	0.7% (n = 2)	0.6% (n = 3)
Baked products, Snacks and sweets	14.9% (n = 43)	10.3% (n = 53)
Vegetables and vegetable products	8.7% (n = 25)	7.5% (n = 39)
*Total Number of Products Coded*	*288*	*517*

Though the proportion of products being promoted was otherwise similar across the remaining categories, it should be noted that low-income stores advertised a higher number of products featured in the following categories: Processed foods and foods containing at least 15 grams of carbohydrates per serving. Circulars from both groups advertised few to no products labeled as low in sodium, reduced-, low-, or zero fat.

#### Comparison of Multiple Sales

Table 2 compares the low- and high-income groups’ proportion of food products within each USDA category that involved a multiple sale. Of the 805 products coded across all the circulars, 32.7% (n = 263) were promotions involving multiple sales. A Chi-square test confirmed that there were no statistically significant differences in this type of promotion between the low- and high-income groups (Chi-Square = 1.173, *p* = 0.279).

This cross-sectional study is limited by the two-month time frame for data collection (May through July, 2013). Also, certain food items advertised may have been seasonal in nature and therefore advertised with greater frequency than in other seasons, e.g., sales promotions for fresh corn. Given the large volume of products for sale in each circular, a single coder was responsible for data collection and only foods on the first page were analyzed. Finally, only digital circulars were analyzed, although it has been noted that digital promotions are about as effective as the printed version with regard to customer preference [26]. We were unable to identify any other studies that looked specifically at promotions in circulars from grocery stores in high- and low-income urban zip codes. 

There were some noted differences in product offerings between high- and low-income groups (Table 1). The grocery stores in low-income zip codes promoted a significantly smaller proportion (approximately half the amount) of products in the USDA category of fruits and fruit juices (10.4% *versus* 20.1%). Additionally, the low-income group offered almost four times as many products (including ketchup, salad dressing and mayonnaise) from the USDA’s food group containing fats, oils, soups, sauces, and gravies. Condiments such as these are considered added sources of calories, saturated fat, sodium, and sugar [27,28]. 

Encouragingly, over one-third (35.9%) of the fresh vegetable products advertised in the low-income group (versus almost one-quarter in the high-income group) were offered as a multiple sale. More than two-thirds of these multiple sales were for fresh corn. This practice of offering multiple sales on seasonal vegetables or fruits encourages increased purchase and consumption of these healthful foods. 

Across both the high- and low-income groups, several similarities are also noteworthy. In both income groups, less than 5% of the products advertised were rich in fiber. Additionally, with 805 products advertised across both groups, there were no low-sodium products and few to no reduced-, low-, or zero fat products offered. As an important measure in preventing heart disease, the leading cause of death among Americans, the USDA and other leading public health agencies recommend a reduction in daily fat and sodium intake and an increased consumption of dietary fiber [29,30]. 

Finally, for both high- and low-income groups, over two-thirds of the products were processed (66.7% and 69.8%) and roughly a quarter contained at least 15g of carbohydrates per serving (24.0% and 27.1%, respectively). Essential to our diet, carbohydrate-containing foods are more beneficial if these foods are also fiber-rich. Although carbohydrate consumption is adequate among Americans, it is of poor nutritional quality [29,30]. Insufficient dietary intake of fiber coupled with excess intake of added sugar and refined grains have contributed to the increased prevalence of chronic diseases [29,30]. 

**Table 2:** Proportion of food products offered with promotions involving multiple sales: Low- *versus* High-income grocery stores in New York City.

Product Characteristics		High-Income			Low-Income	
	USDA Food Group	Proportion of USDA Food Group that Meets Product Characteristics	Proportion of Promotions involving Multiple Sales	USDA Food Group	Proportion of USDA Food Group that Meets Product Characteristics	Proportion of Promotions involving Multiple Sales
Baked products containing ≥ 15 g carbohydrates/serving	Baked products (N = 20)	75% (n = 15)	50% (n = 10)	Baked products (N = 19)	89.5% (n = 17)	5.3% (n = 1)
Baked products containing ≥ 5 g fiber/serving	Baked products (N = 20)	0% (n = 0)	0% (n = 0)	Baked products (N = 19)	0% (n = 0)	0% (n = 0)
Breakfast cereals containing ≥ 15 g carbohydrates/serving	Breakfast cereals (N = 3)	100% (n = 3)	0% (n = 0)	Breakfast cereals (N = 10)	100% (n = 10)	10% (n = 1)
Breakfast cereals containing ≥ 5 g fiber/serving	Breakfast cereals (N=3)	0% (n = 0)	0% (n = 0)	Breakfast cereals (N = 10)	0% (n = 0)	0% (n = 0)
Cereal grains and pasta products containing ≥ 15 g carbohydrates/serving	Cereal grains and pasta products (N = 1)	100% (n = 1)	0% (n = 0)	Cereal grains and pasta products (N = 34)	100% (n = 34)	29.4% (n = 10)
Cereal grains and pasta products containing ≥5 g fiber/serving	Cereal grains and pasta products (N = 1)	0% (n = 0)	0% (n = 0)	Cereal grains and pasta products (N = 34)	17.5% (n = 6)	11.8% (n = 4)
Fresh fruits	Fruits and fruit juices (N = 58)	94.8% (n = 55)	24.1% (n = 14)	Fruits and fruit juices (N = 54)	81.5% (n = 44)	18.5% (n = 10)
Canned/frozen fruits	Fruits and fruit juices (N = 58)	0% (n = 0)	0% (n = 0)	Fruits and fruit juices (N = 54)	0% (n = 0)	0% (n = 0)
Fruit juices	Fruits and fruit juices (N = 58)	5.2% (n = 3)	3.4% (n = 2)	Fruits and fruit juices (N = 54)	18.5% (n = 10)	3.7% (n = 2)
Fresh vegetables	Vegetable and vegetable products (N = 25)	24.0% (n = 6)	24.0% (n = 6)	Vegetable and vegetable products (N = 39)	59.0% (n = 23)	35.9% (n = 14)
Canned/frozen vegetables	Vegetable and vegetable products (N = 25)	76.0% (n = 19)	24.0% (n = 6)	Vegetable and vegetable products (N = 39)	41.0% (n = 16)	15.4% (n = 6)

## 4. Conclusions

Our current understanding from the literature is that populations in lower-income communities have less access to nutritious foods. Our study’s findings are contradictory in that the proportions of healthful and unhealthful foods advertised on the front page of grocery store circulars were similar across both low- and high-income groups. There are important public health implications to these findings. Circulars, used as a means to market sale items, can be considered as part of the built environment which has been described in the literature as an important factor in shaping health-related behaviors [31].

According to a recent marketing report, grocery stores’ use of print circulars is increasing [13]. A 2011 report indicated that of the 70% of shoppers who make a list prior to going to the grocery store, almost half utilize circulars in making that list [32]. In addition, coupon use is on the rise which may be a marker of the recent economic recession and resultant financial constraints of customers [33]. 

In low-income urban areas, accessibility to healthy foods is greatly diminished amid the large number of smaller establishments such as bodegas and delis that generally offer foods and beverages lower in nutritional value. Grocery stores in these areas can utilize circulars as an important mechanism to promote affordable, nutritious foods. In our study, the circulars from stores located in low-income zip codes collectively advertised over 500 products on their front pages thereby highlighting a demand for foods priced to sell. Clearly, a strategic opportunity exists to offer more healthful foods at an affordable price to a population that is at higher risk for nutrition-related chronic disease.

Grocery stores have begun to implement more innovative efforts to promote the health of their customers. One Indiana-based chain has instituted both in-store programs supervised by a registered dietician as well as online resources and tools to increase customers’ nutrition-related knowledge and purchases [34]. A program focusing on diabetes care was also recently instituted by this chain and promotes more healthful eating through various strategies including online support from a dietician, video cookbook, and enrollment in an educational program, all at no cost [35]. 

In addition to initiatives like these, more traditional channels such as weekly circulars can be adapted to increase the availability and highlight placement of more nutritious foods on a regular basis. For instance, advertisements for seasonal fruits and vegetables can be regularly and prominently placed in circulars with a focus on multiple sales, e.g., two cartons of berries for the price of one or reducing the cost per pound of seasonal vegetables. 

The strategy of cross-promotion encourages customers to purchase two or more products associated in some manner, e.g., ice cream and chocolate syrup or pasta and meat sauce. This method has been suggested as a store-based strategy to increase the promotion and sale of nutritious foods [36] and could also be utilized in circulars with prominent placement of such pairings as fresh fruit and yogurt, brown rice and beans, or fresh produce with low-fat salad dressing. One grocery store chain has developed a circular exclusively devoted to nutritious foods and includes this as part of a larger health promotion effort. The circular utilizes cross-promotion, additional coupons, recommendations from the chain’s registered dietician, nutrition information on products offered, and additional web-based resources [37]. 

Given their documented, extensive reach, circulars should be considered a potentially effective medium to promote the purchase and consumption of healthy foods. While it is known that advertising and marketing strategies are successful at influencing behaviors, public health research and program planning efforts can focus on partnering with grocery stores to incorporate more prominently placed, nutritious food choices on weekly circulars. Aligned nutrition education efforts can focus on supporting customers in identifying those products and promotions that are beneficial to their health. 

## References

[1] Larson N., Story M., Nelson M. (2009). Neighborhood environments: Disparities in access to healthy foods in the U.S. Am. J. Prev. Med..

[2] Andreyeva T., Luedicke J., Middleton A., Long M., Schwartz M. (2011). Changes in Access to Healthy Foods after Implementation of the WIC Food Package Revisions.

[3] PolicyLink The Grocery Gap: Who Has Access to Healthy Foods and Why It Matters. http://thefoodtrust.org/uploads/media_items/grocerygap.original.pdf.

[4] Robert Wood Johnson Foundation Bringing Healthy Foods Home: Examining Inequalities in Access to Food Stores. http://www.healthyeatingresearch.org/images/stories/her_research_briefs/her%20bringing%20healthy%20foods%20home_7-2008.pdf.

[5] Salois M.J. (2012). Obesity and diabetes, the built environment, and the “local” food economy in the United States, 2007. Econ. Hum. Biol..

[6] Herrera N.M., Herrera N.M., United States Department of Agriculture (2011). Access to Affordable and Nutritious Food: Measuring and Understanding Food Deserts and Their Consequences. Eating Right: The Consumption of Fruits and Vegetables.

[7] Zick C., Smith K.R., Fan J.X., Brown B.B, Yamada I., Kowaleski-Jones L. (2009). Running to the store? The relationship between neighborhood environments and the risk of obesity. Soc. Sci. Med..

[8] Morland K., Diez-Roux A., Wing S. (2006). Supermarkets, other food stores, and obesity: The atherosclerosis risk in communities study. Am. J. Prev. Med..

[9] Rose D., Richards R. (2004). Food store access and household fruit and vegetable use among participants in the US Food Stamp Program. Public Health Nutr..

[10] Ahern M., Brown C., Dukas S. (2011). A national study of the association between food environments and county-level health outcomes. J. Rural Health.

[11] Glanz K., Sallis J.F., Saelens B.E., Frank L.D. (2007). Nutrition environment measures survey in stores (Nems-S): Development and evaluation. Am. J. Prev. Med..

[12] Food Trends Get Technical, Sustainable & Healthy. http://www.forbes.com/sites/daniellegould/2012/12/28/2013-food-trends-get-technical-sustainable-healthy/.

[13] Use of Print Circulars Grows in 2011. http://supermarketnews.com/datasheet/january-30-2012-use-print-circulars-grows-2011.

[14] Eat Smart. http://www.bashas.com/healthyliving/EatSmart.aspx.

[15] Ethan D., Samuel L., Basch C.H. (2013). An analysis of Bronx-based online grocery store circulars for nutritional content of food and beverage products. J. Commun. Health.

[16] Samuel L., Basch C.H., Ethan D., Hammond R., Chiazzese K. (2013). An analysis of sodium, total fat and saturated fat contents of packaged food products advertised in Bronx-based online supermarket circulars. Am. J. Hypertens..

[17] Algert S.J., Agrawal A., Lewis D. (2006). Disparities in access to fresh produce in low-income neighborhoods in Los Angeles. Am. J. Prev. Med..

[18] Zenk S.N., Schulz A.J., Israel B.A., James S.A., Bao S., Wilson M.L. (2006). Fruit and vegetable access differs by community racial composition and socioeconomic position in Detroit, Michigan. Ethn. Dis..

[19] Powell L., Slater S., Mirtcheva D., Bao Y., Chaloupka F. (2007). Food store availability and neighborhood characteristics in the United States. Prev. Med..

[20] United States Census Bureau Homepage. http://www.census.gov/.

[21] USDA National Nutrient Database for Standard Reference Release 26. http://ndb.nal.usda.gov/ndb/search/list.

[22] Brown M.D., Lackey H.D., Miller T.K., Priest D. (2001). Controlling calories—the simple approach. Diabet. Spectr..

[23] Anderson J., Perryman S., Young L., Prior S. Dietary Fiber Fact.

[24] Sodium in Your Diet. http://www.fda.gov/downloads/Food/IngredientsPackagingLabeling/UCM315471.pdf.

[25] The Significance of the Difference Between Two Independent Proportions. http://www.vassarstats.net/propdiff_ind.html.

[26] Digital Coupons Rival Print Counterparts in Effectiveness. http://www.emarketer.com/Article/Digital-Coupons-Rival-Print-Counterparts-Effectiveness/1008982.

[27] Healthier Condiments. http://www.heart.org/HEARTORG/GettingHealthy/NutritionCenter/HealthyCooking/Healthier-Condiments_UCM_445176_Article.jsp.

[28] Guidelines for A Low Cholesterol, Low Saturated Fat Diet. http://www.ucsfhealth.org/education/guidelines_for_a_low_cholesterol_low_saturated_fat_diet/.

[29] Dietary Guidelines for Americans, 2010. http://health.gov/dietaryguidelines/dga2010/dietaryguidelines2010.pdf.

[30] Lifestyle Changes and Cholesterol. http://www.heart.org/HEARTORG/Conditions/Cholesterol/PreventionTreatmentofHighCholesterol/Lifestyle-Changes-and-Cholesterol_UCM_305627_Article.jsp.

[31] Booth K.M., Pinkston M.M., Poston W.S. (2005). Obesity and the built environment. J. Am. Diet. Assoc..

[32] CPG 2011 Year in Review: The Search for Footing in An Evolving Marketplace. http://www.iriworldwide.com/portals/0/articlePdfs/T_T%20February%202012%20Presentation.pdf.

[33] Glanz K., Bader M.D., Iyer S. (2012). Retail Grocery store marketing strategies and obesity: An integrative review. Am. J. Prev. Med..

[34] (2013). Marsh Tackles Obesity with New Wellness Offerings. http://www.progressivegrocer.com/top-stories/headlines/health-wellness/id33872/marsh-tackles-obesity-with-new-wellness-offerings/.

[35] Diabetes Care. http://www.marsh.net/pharmacy/diabetes-care/.

[36] Glanz K., Yaroch A.L. (2004). Strategies for increasing fruit and vegetable intake in grocery stores and communities: Policy, pricing, and environmental change. Prev. Med..

[37] Bashas’ Snack smart!. http://www.bashas.com/PDF/eatSmart20130916.pdf.

